# Literary Notes

**Published:** 1909-06

**Authors:** 


### LITERARY NOTES

The philosophy of long life is interesting to all who believe in the happiness of health, or who are interested in the Emmanuel Movement, or the theories of Metchnikoff and Fletcher. The "Philosophy of Long Life," published this week by John Lane Company, should be a stimulus to all such. Death and life have been the two subjects which have commanded the attention of mankind more than all else from the building of the pyramids to the present. M. Finot looks at life as a force which permeates all things, inanimate objects as well as animals. His conclusion, therefore, is that life is indestructible, and he gives practical suggestions for the prolongation of our life in this world, and for banishing the helplessness of old age. He believes that he has found "The Cure for Old Age," and teaches calmness in the face of death. The book has run through fourteen editions in France alone, and is being translated into almost every language. It is a philosophy for lovers of life.

---

The Modern Medicine Publishing Co., announce for early publication a work on phototherapy entitled Light Therapeutics, a practical manual: physics, physiologic effects, technic, therapeutics, clinical applications, by Dr. J. H. Kellogg, Superintendent of the Battle Creek Sanitarium. Light Therapeutics will contain about 225 pages and 75 illustrations, printed in large clear type, and substantially bound in cloth. Price, probably, $2.00 net.

---

*Journal of Pharmacology and Experimental Therapeutics* is announced. Without rigidly defining the scope of the new journal it may be stated that its pages will be open not only to pharmacologists and workers in experimental therapeutics but also to the representatives of any of the biological or medical sciences who may wish to offer papers that have a close relationship to pharmacological or therapeutical questions.

The Journal will be carried on with the collaboration of the members of the recently organised Society for Pharmacology and Experimental Therapeutics.

In addition, a number of leading men of science in this country and in Europe have promised the new journal their support.

At least six numbers of the Journal will be issued yearly and will constitute a volume of not less than six hundred or more than six hundred and fifty pages.

The price of the Journal will be $5.00 per volume, sent postpaid to subscribers in all countries. Contributors will receive one hundred copies of their papers free of charge and additional copies will be furnished at cost.

The first number of the Journal will be issued at an early date—about May 1, 1909.

Subscriptions and correspondence concerning business matters should be addressed to The Williams & Wilkins Publishing Company, 2427-2429 York Road, Baltimore. Specimen copies of the Journal will be supplied upon application to the publishers.

